# Enhancement of the Molecular and Serological Assessment of Hepatitis E Virus in Milk Samples

**DOI:** 10.3390/microorganisms8081231

**Published:** 2020-08-12

**Authors:** Ibrahim M. Sayed, Ahmed R. A. Hammam, Mohamed Salem Elfaruk, Khalid A. Alsaleem, Marwa A. Gaber, Amgad A. Ezzat, Eman H. Salama, Amal A. Elkhawaga, Mohamed A. El-Mokhtar

**Affiliations:** 1 Department of Medical Microbiology and Immunology, Faculty of Medicine, Assiut University, Assiut 71515, Egypt or i4ibrahim@health.ucsd.edu (I.M.S.); Amy.elkhawaga@yahoo.com (A.A.E.); 2 Department of Pathology, School of Medicine, University of California, San Diego, La Jolla, CA 92093, USA; 3 Dairy and Food Science Department, South Dakota State University, Brookings, SD 57007, USA; ahmed.hammam@sdstate.edu (A.R.A.H.); Mohamed.elfaruk@sdstate.edu (M.S.E.); Khalid.alsaleem@sdstate.edu (K.A.A.); 4 Dairy Science Department, Faculty of Agriculture, Assiut University, Assiut 71526, Egypt; 5 Medical Technology College, Nalut University, Nalut 00218, Libya; 6 Department of Food Science and Human Nutrition, College of Agriculture and Veterinary Medicine, Qassim University, Buraydah 51452, Saudi Arabia; 7 Department of Medical Biochemistry, Faculty of Medicine, Assiut University, Assiut 71515, Egypt; marwagaber@aun.edu.eg; 8 Department of Microbiology and Immunology, Faculty of Medicine, Al-Azhar University, Assiut 71524, Egypt; amgadezzat@azhar.edu.eg; 9 Department of Clinical Pathology, Faculty of Medicine, Sohag University, Sohag 82524, Egypt; eman_salama@med.sohag.edu.eg

**Keywords:** HEV, milk, molecular assay, RNA extraction, serology, skim milk, milk serum, virus recovery, limit of detection.

## Abstract

Hepatitis E virus (HEV) infection is endemic in developing and developed countries. HEV was reported to be excreted in the milk of ruminants, raising the possibility of transmission of HEV infection through the ingestion of contaminated milk. Therefore, the detection of HEV markers in milk samples becomes pivotal. However, milk includes inhibitory components that affect HEV detection assays. Previously it was reported that dilution of milk matrix improves the performance of HEV molecular assay, however, the dilution of milk samples is not the best strategy especially when the contaminated milk sample has a low HEV load. Therefore, the objective of this study is to compare the effect of extraction procedures on the efficiency of HEV RNA detection in undiluted milk samples. In addition, we assessed the effect of the removal of milk components such as fats and casein on the performance of the molecular and serological assays of HEV. Phosphate buffered saline (PBS) and different milk matrices (such as whole milk, skim milk, and milk serum) were inoculated with different HEV inoculums and subjected to two different extraction procedures. Method A includes manual extraction using spin column-based extraction, while method B includes silica-based automated extraction. Method A was more sensitive than method B in the whole milk and skim milk matrices with a LoD_95%_ of 300 IU/mL, and virus recovery yield of 47%. While the sensitivity and performance of method B were significantly improved using the milk serum matrix, with LoD_95%_ of 96 IU/mL. Interestingly, retesting HEV positive milk samples using the high sensitivity assay based on method B extraction and milk serum matrix increased the HEV RNA detection rate to 2-fold. Additionally, the performance of HEV serological assays such as anti-HEV IgG and HEV Ag in the milk samples was improved after the removal of the fat globules from the milk matrix. In conclusion, HEV RNA assay is affected by the components of milk and the extraction procedure. Removal of inhibitory substances, such as fat and casein from the milk sample increased the performance of HEV molecular and serological assays which will be suitable for the low load HEV milk with no further dilutions.

## 1. Introduction

Hepatitis E virus (HEV) is the most causative agent for acute viral hepatitis globally. It infects about 20 million people resulting in 70000 deaths annually [1]. HEV is a positive sense single-strand RNA virus that belongs to the *Orthoherpesvirus* genus of the *Herpesviridae* family [2,3]. The *Orthoherpesvirus* A includes eight genotypes (gt) (HEV-1-8). HEV-1 and HEV-2 are transmitted by the fecal–oral route, and they are mainly associated with waterborne HEV outbreaks in developing countries [4,5]. HEV-3 and HEV-4 infect humans and animals such as pigs, wild boars, deer, rabbits, cows, and goats [6,7]. They are zoonotic, infection is mainly transmitted to humans either by contact with these animals or the ingestion of undercooked meat, sausages, liver, or other products from them [8,9,10]. Additionally, HEV-3 and HEV-4 were reported to be excreted in the milk of ruminants [11,12,13,14]. HEV particles excreted in milk were infectious to in vivo animal models and they could be inactivated by short time boiling but not by pasteurization [12]. HEV-7 and HEV-8 were identified in camels [15,16], HEV-7 is also zoonotic, chronic HEV infection was documented in a liver transplant patient through the frequent ingestion of the camel meat and milk (camelid HEV-7), raising the possibility of HEV transmission via the contaminated milk [17]. Other HEV isolates such as HEV-5, HEV-6, and HEV-8 have not been confirmed as human pathogens [3,18].

Our group recently reported the detection of HEV in the milk of the cows and goats distributed in rural communities of Assiut governorate, Egypt. In these previous reports, we used a molecular qRT-PCR assay targeting the ORF2/3 region, which is highly conserved among all HEV genotypes, to detect HEV RNA in the milk samples. The LoD_95_ % (LoD: limit of detection, which is the lowest HEV RNA concentration that could be detected in 95% of replicates) of this assay was 300 IU/mL [13,14].

The composition of food products can affect the process of enterovirus extraction and the recovery of the viral nucleic acid from the matrix [19,20,21,22]. The detection of enteric viruses in food is challenging due to the low level of viral contamination in the food and the presence of substances that can inhibit PCR amplification [23,24]. Milk is composed of different components such as fats, casein, whey proteins, and lactose that have different characteristics including adsorption, sedimentation, and separation. Several reports showed that the milk matrix and milk components affect the process of viral nucleic acid extraction and viral recovery yield [20,25,26,27]. Additionally, different processing and extraction procedures affect the detection and recovery of enteric viruses such as HAV and norovirus (NoV) from the milk matrix [20,25]. It was reported that the milk matrix affected the sensitivity and performance of a closed automated system (The cobas^®^ 6800 system (Roche Diagnostics) that detects HEV RNA (gt1-4) in the human plasma with a LoD_95_% of 18.6 IU/mL. To reduce the inhibitory substances in the milk without affecting the assay performance, milk samples were diluted with PBS to form a 10% diluted sample [28]. However, dilution of milk samples (10%) would not be the best strategy, especially if the viral load in the original undiluted samples is low. Moreover, the highly sensitive cobas^®^ 6800 system is expensive and not available all over the world. Previous reports showed that the HEV load was comparable in the milk and serum samples and high HEV titer was detected in the milk of humans, cows, and goats [12,29,30]. However, our previous studies illustrated that the HEV load excreted in the milk was relatively low (around 10^3^ IU/mL), probably the difference in circulating virus, geographic distribution, mixing of ruminants with other HEV reservoirs, etc., could explain this discrepancy [13,14]. Therefore, we attempted to improve the sensitivity and performance of the HEV molecular assays without diluting the original samples.

To date, there is no study that describes the effect of extraction procedures and milk components on the HEV molecular assays. Herein, we compared the effect of different extraction procedures on the sensitivity of HEV molecular assay, as well as, we assessed the impact of milk components on the performance of HEV molecular and serological assays. Finally, we evaluated the efficiency of these optimizing procedures on milk samples collected from HEV positive cows and goats distributed in Egyptian villages.

## 2. Materials and Methods

### 2.1. HEV Inoculum Used in the Study

HEV-3 inoculum was isolated from the stool of acute HEV-infected patient admitted for diagnostic purposes to Assiut University hospitals (Assiut, Egypt) as described previously [31,32]. Briefly, the stool was weighed, and then a 10% *w/v* fecal suspension was prepared in PBS. The fecal suspension was centrifuged, and the supernatant was collected and filtered using a 0.22-μm filter (Millipore, MA, USA). The viral load of this inoculum was quantified by qRT-PCR, as described previously [14]. Briefly, the World Health Organization (WHO) International Standard (IS) containing HEV reference preparation code number 6329/10 (Paul Ehrlich Institute, Langen, Germany) of initial concentration 250,000 IU/mL was used [33]. Serial dilutions of the reference were done in PBS, and qRT-PCR was performed using the primers and probe targeting HEV ORF2/3, as will be described in detail below. Each standard was tested in triplicate. According to the cycle threshold (Ct), a standard curve was generated. HEV-3 inoculum was run at the same time with the standards, and according to the Ct of HEV inoculum, the viral load was calculated. Serial dilutions of stool derived HEV-3 (3 × 10^5^–30 IU/mL) were added to PBS and cow milk matrix (previously evaluated, negative to all HEV markers). VetMAX™ Xeno™ Internal Positive Control (IPC) (conc 10,000 copies/uL) (ThermoFischer Scientific, Grand Island, NY, USA) was added to each dilution.

### 2.2. Processing of the Milk Samples and Separation of the Milk Components

We used in this study whole milk, skim milk, and milk serum matrices. The skim milk and milk serum were prepared as described previously with a slight modification in the procedure [25,26,34,35]. The skim milk was prepared by centrifugation of the whole milk sample at 4000× *g* for 30 min at 4 °C to remove the cream layer that includes mainly the fat globules. The milk serum was prepared by centrifugation of the skim milk at 50,000× *g* for 6 h to precipitate the casein micelles and separate the milk serum.

### 2.3. RNA Extraction Methods

PBS and different milk matrices inoculated with different HEV inoculums were subjected to two different extraction procedures

**Method A:** manual extraction was performed using column-based extraction methods via two commercially available kits; QIAamp^®^ Viral RNA kit (Qiagen, Hilden, Germany), and High Pure Viral Nucleic Acid Kit (Roche LifeScience, Upper Bavaria, Germany) according to the manufacturer’s instructions. Both the kits involve the spin column, washing steps, and centrifugation steps. The first kit utilizes a silica-based column, and the second kit utilizes a glass fiber fleece spin filter tube. The initial sample volume was 200 μL, and the elution volume was 50 μL.

**Method B:** includes the NUCLISENS easyMAG extraction (bioMérieux, Marcy Letolle, France) using magnetic silica beads as described previously [36,37,38] and according to the manufacturer’s instructions. The initial sample volume was 200 μL, and the elution volume was 50 μL.

PBS sample and milk matrices without HEV inoculums were extracted by the previous two methods as negative control samples. An Internal Positive Control (IPC) was added to each sample as a control for the extraction efficiency.

### 2.4. HEV RNA qRT-PCR

Detection and quantification of HEV RNA in the extracted RNA samples were done using primers and probes that target ORF2/3 [39]. Briefly, RNA samples were converted into cDNA using RevertAid First Strand cDNA Synthesis Kit (ThermoFischer Scientific, Waltham, NY, USA) according to the manufacturer’s instructions. Amplification was performed using TaqMan™ Universal PCR Master Mix (ThermoFischer Scientific, Waltham, NY, USA) on the Applied Biosystems 7500/7500 Fast (Applied Biosystems, Foster City, CA, USA) following the condition suggested by the manufacturer’s instructions. The following primers and probe targeting the HEV ORF2/ORF3 overlapping region were used: forward primer HEV ORF2/ORF3-S: 5′-GGTGGTTTCTGGGGTGAC-3′, reverse primer HEVORF3-AS: 5′-AGGGGTTGGTTGGATGAA-3′ and probe: 5′-FAMTGATTCTCAGCCCTTCGC-TAMRA-3′. For RNA process control (IPC), the qPCR was done using the primer and probe provided in the kit according to the manufacturer’s instructions. Quality control of the RT-qPCR process included negative (nuclease-free water) and positive (HEV RNA) controls added to each PCR plate. Each sample was analyzed in duplicate/run. The estimated probability of detection with 95% confidence (LoD_95%_) was calculated by using the PODLOD calculation program (version 9) [40]

### 2.5. Evaluation of the Effect of Extraction Procedures and Milk Components on Potential HEV Positive Milk Samples

**Figure 1:**
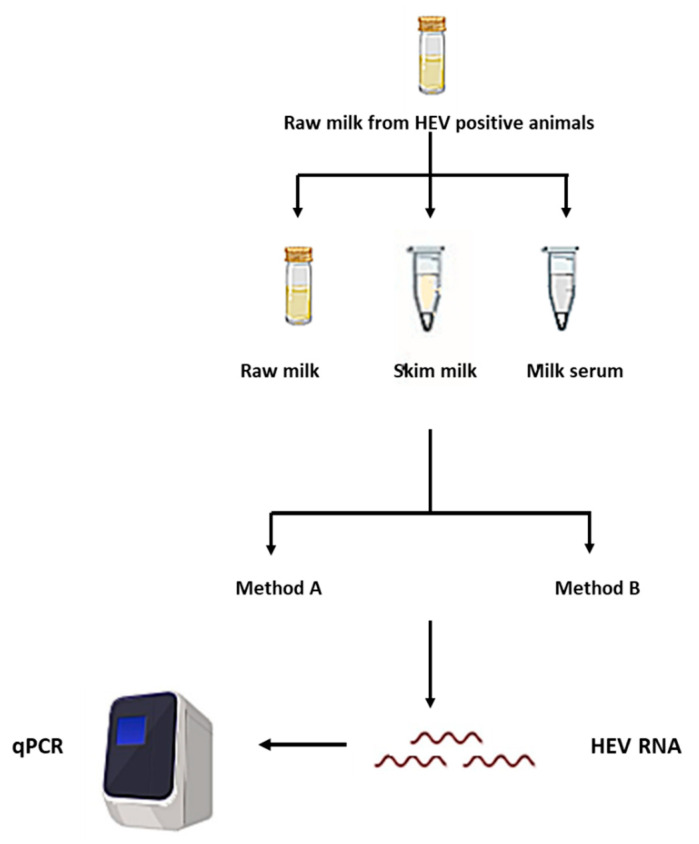
A schematic diagram showing the study design of the effect of extraction procedures and milk components on hepatitis E virus (HEV) molecular assays.

Recently we reported HEV markers in the cow and goat milk samples distributed in the Egyptian villages [13,14]. In potential HEV infected milk samples, we previously detected HEV RNA in three samples (one cow milk and two goat milk) out of 28 samples (10.7%) that were anti-HEV IgG positive. We did the nucleic acid extraction process using method A (QIAMP Viral RNA kit) on the whole milk matrix and our LoD_95%_ was 300 IU/mL. The HEV load in the milk samples was low (around 10^3^ IU/mL). Since we found that the sensitivity of the HEV molecular assays could be improved using method B and milk serum matrix, we retested those samples again after the removal of milk components and using the methods described previously (Figure 1)

### 2.6. Detection of Anti-HEV IgG in the Milk Samples

Detection of anti-HEV IgG in the cow and goat milk samples was performed as described previously [13,14]. Milk samples collected from healthy non-infected cows and goats served as negative control samples. We used an adapted protocol of commercial ELISA kit (Wantai Biologic Pharmacy Enterprise, Beijing, China) as described previously with a slight modification in the procedure [13,14,28]. We replaced the kit’s conjugate antibody (Horseradish peroxidase-conjugated rabbit anti-human IgG antibody) with an HRP-conjugated rabbit anti-goat IgG antibody (Sigma-Aldrich, St. Louis, MO, USA) or with HRP-conjugated rabbit anti-bovine IgG antibody (Sigma-Aldrich, St. Louis, MO, USA) for the detection of anti-HEV IgG in the goat and cow milk samples, respectively. Positive and negative control (NC) from kits used directly and/or after soaking in HEV negative milk samples (cow/goat) were used as control.

**Figure 2:**
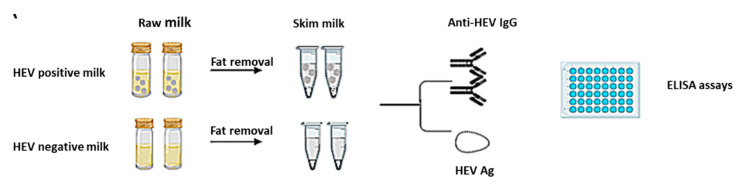
Schematic design showing the experiment design “Effect of fat removal on the performance of HEV serology assay.”

The cut-off = the average OD of the triplicate NC from the kit soaked in HEV negative animal (goat/cow) milk + 3*standard deviation. For comparison between whole milk and skim milk (Figure 2), the cut off was recalculated according to the equation mentioned after soaking of HEV negative control from the kit in those milk matrices.

### 2.7. Detection of HEV Ag in Goat and Cow Milk Samples

HEV ORF2 Ag was assessed in the goat and cow milk samples using the HEV-Ag ELISA^Plus^ assay (Bejing Wantai Biological Pharmaceutical Co., Beijing, China) according to the manufacturer’s instructions, with slight modifications in the procedure of cut-off (C.O.) calculation as described previously [13,14,41]. For comparison between whole milk and skim milk (Figure 2), the cut off was recalculated according to the equation mentioned after soaking of the HEV negative control from the kit in those milk matrices.

### 2.8. Statistics

Statistical analyses were performed using the GraphPad Prism software 6 (GraphPad Software, La Jolla, CA, USA) using Student’s *t*-test and Tukey’s multiple comparison tests. *p* < 0.05 was considered significant.

## 3. Results

### 3.1. HEV Molecular Assay is affected by the Milk Matrix

**Figure 3:**
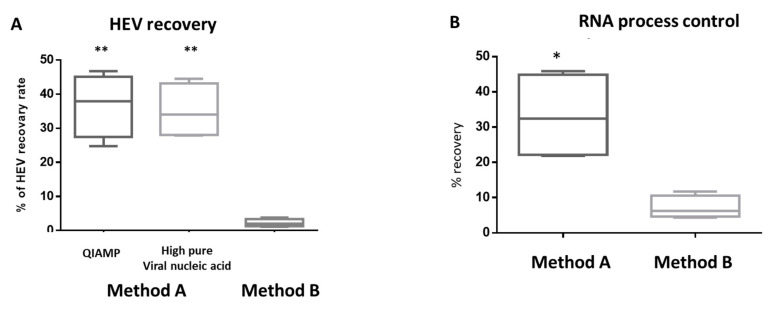
(**A**) Comparing the effect of two extraction procedures on the recovery of HEV from the whole milk matrix. Method A includes spin column-based manual extraction, and method B includes automated extraction using magnetic beads. Comparison of HEV recovery in method A vs. method B, ** indicates *p* < 0.01 as determined by unpaired two-tailed Student’s *t*-test. (**B**) The recovery yield of Internal Positive Control (IPC) added to each sample was calculated for the two extraction procedures. Method A represented here includes data of high pure viral nucleic acid. * indicates *p* < 0.05 as determined by unpaired Student’s *t*-test.

In the PBS matrix, the automated extraction showed superior results to the column-based manual extraction. The LoD_95%_ of method B (automated extraction) was 19.4 IU/mL and 12.9 copies/mL for HEV RNA and IPC, respectively. While the LoD_95%_ of method A was 96.6 IU/mL and 64.2 copies/mL for HEV RNA and IPC, respectively. We did not find a difference between the two commercial extraction kits of the method A used. In the whole milk matrix, manual RNA extraction (method A) was more efficient than method B regarding sensitivity and virus recovery. The LoD_95%_ of method A was 300 IU/mL and 651 copies/mL for HEV RNA and IPC, respectively. In contrast, LoD_95%_ of method B was 6.12 × 10^3^ IU/mL and 1412.3 copies/mL for HEV RNA and IPC, respectively (Table 1). The average HEV recovery yield from the whole milk matrix using method A was about 36.8% and 35% for QIAamp Viral RNA Kit and High pure viral nucleic acid kit, respectively. While the average HEV recovery yield from the whole milk matrix using method B was about 2.2% (Figure 3).

Then we asked if the presence of inhibitory substances in the RNA extracted by method B from the milk matrix affects the PCR performance. Both undiluted RNA and 1/10 diluted RNA from the virus dilution 3 × 10^5^–3 × 10^3^ IU/mL were retested for HEV RNA using the same PCR assay. The HEV viral load was quantified, and the virus recovery rate was calculated. Importantly, we did not notice a significant difference in the virus recovery rate between undiluted, and 1/10 diluted RNA and the difference in cycle threshold (Ct) value between the two preparations was about three cycles indicating the absence of PCR inhibitors in the RNA extract obtained from method B (Table 2).

Collectively, results from Table 1 and Table 2 suggest that the extraction procedure and matrix affect the HEV RNA detection rate from the whole milk matrix. Since the two commercial extraction kits of method A showed the same performance, we decided to complete the study using one extraction kit (high pure viral nucleic acid).

### 3.2. Milk Components Affect the Performance of HEV Molecular Assay

**Figure 4:**
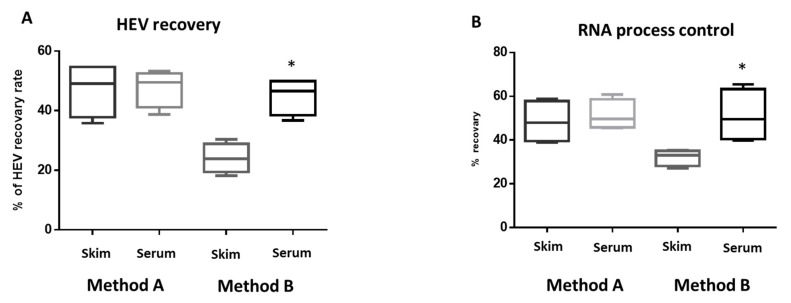
(**A**) Comparing the effect of extraction procedures and milk matrices on the recovery of HEV from the whole milk matrix. Comparison of HEV recovery in skim milk matrix and milk serum matrix using method B, * indicates *p* < 0.05 as determined by Tukey’s multiple comparison tests. (**B**) The recovery yield of IPC. Comparison of IPC recovery in the skim milk matrix and milk serum matrix using method B * indicates *p* < 0.05 as determined by Tukey’s multiple comparison tests.

To assess the effect of milk components on the HEV molecular assay, serial dilutions of the stool derived HEV-3 inoculums (3 × 10^5^–30 IU/mL) were added to the skim milk matrix (without fat globules) and milk serum matrix (without fat globules and casein micelles). Then, we assessed LoD_95%_ and the virus recovery yield using the previous methods and compared these values with the whole milk matrix (Table 1). Method A resulted in LoD_95%_ of 300 IU/mL for HEV RNA for the skim milk and skim serum matrices, which were the same LoD_95%_ of whole milk matrix. While the HEV recovery rate was higher in the skim milk matrix (47.17 ± 7.95) and milk serum matrix (47.47 ± 5.45) compared to the whole milk matrix (35.1 ± 7.03) (Figure 3 and Figure 4). The recovery yield and LoD_95%_ of IPC were improved in the skim milk and milk serum matrices compared to the whole milk matrix (Table 3, Figure 3 and Figure 4). Using method B, both the LoD95% and the recovery yield of HEV and IPC were improved in the skim milk matrix. The LoD_95%_ became 977 IU/mL, and the virus recovery rate increased about 5–10 times compared to the HEV yield from the whole milk matrix. Importantly, lower LoD_95%_ (96 IU/mL) and higher HEV recovery rate (45 ± 5.43) were achieved using the method B extraction of HEV from the milk serum matrix (Table 3, Figure 4). Similarly, the performance of IPC processed by method B was improved significantly in the milk serum compared to the skim milk matrix.

### 3.3. Evaluation of the Effect of Extraction Procedures and Milk Components on Potential HEV Positive Milk Samples

Using method A, we got the same results in whole milk, skim milk, and milk serum (10.7%). Using method B, we could not detect HEV RNA in any of the tested whole milk samples. While we could detect HEV RNA in two out of 24 milk samples in the skim milk matrix (7.14%). Interestingly, we detected six out of 24 milk samples (21.4%) in the milk serum matrix using method B. The HEV load in the milk samples that could not be detected by method A but detected by method B in milk serum matrix ranged from 1.68 × 10^2^ to 2.5 × 10^2^ IU/mL (Table 4), which was lower than the LoD_95_% of method A.

### 3.4. Removal of the Fat Globules Increased the Performance of Anti-HEV IgG and HEV Ag Assays in the Milk Samples

**Figure 5:**
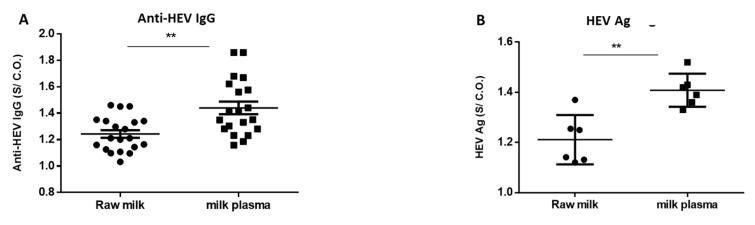
Effect of fat removal on the performance of HEV serology assay. (Anti HEV IgG (**A**) and HEV Ag (**B**) were retested in the cow and goat milk samples before (whole milk) and after (skim milk) the removal of fat globules. Negative whole milk samples and negative skim samples were run in the same assay, and the cut off was calculated. S/C.O. means absorbance/ cut off. ** indicates *p* < 0.01 as determined by unpaired Student’s *t*-test.

HEV positive milk samples (n = 20) and HEV negative milk samples (n = 20) were analyzed for HEV markers before and after the removal of the fat globules (Figure 2). We found that the absorbance of anti-HEV IgG (S/C.O.) was significantly improvedin the milk samples after the removal of the fat globules (Figure 5A). Three milk samples with S/C.O. were at the grey zone: 1.097, 1.03, and 1.095. After the removal of fat globules and reassessment of these samples, we found that the S/C.O. became 1.23, 1.18, and 1.62, respectively.

Regarding HEV Ag, only six samples were positive for HEV Ag. We assessed the effect of the removal of the fat globules on the detection of HEV Ag. Similar to anti-HEV IgG, the absorbance of HEV Ag was significantly increased after the removal of the fat globules (Figure 5B).

## 4. Discussion

Foodborne infections caused by viral pathogens such as HEV represent an important global public health issue. The European Food Safety Authority (EFSA) recently pointed foodborne viruses and highlighted HEV among the topics of the food safety regulatory research needs 2030 [42]. HEV foodborne infection is mainly transmitted by the ingestion of contaminated/undercooked food products such as meat, liver, and sausages derived from pigs, wild boars, and deer [8,17,43,44]. Additionally, the HEV outbreak associated with shellfish consumption was reported [45]. Recently, HEV was detected in the milk of cows and goats [11,12,14,30]. This data suggests the possibility of HEV transmission through the ingestion of contaminated milk. People living in the villages and rural communities are at high risk due to close contact with the animals and consumption of unpasteurized milk is a traditional meal. Furthermore, regions that were reported endemic for HEV with high HEV prevalence rate and close homology between HEV isolates circulating between human and animals could be also at high risk for acquisition of HEV infection through the ingestion of the dairy products. Some evidence could directly or indirectly support the risk of HEV transmission through the ingestion of contaminated milk products. For example, chronic HEV was diagnosed in a liver transplant patient who regularly consumed camel meat and milk [17]. Additionally, we recently reported that 80% of the Egyptian rural households, who owned seropositive goats and regularly consumed the goat dairy products, were also HEV seropositive [13]. Likewise, Mesquita et al. reported that the risk of HEV infection is increased in shepherds and sheep milk cheesemaker-workers in Portugal [46]. Furthermore, HEV contaminated cow milk products were infectious to in vivo animal models [12]. Finally, there is a close phylogenetic relationship between human and HEV strains isolated from the milk samples [11,12,30]. All these data confirm that contaminated milk is a potential risk source for HEV infection. Therefore, the assessment of HEV in the milk becomes crucial for consumers’ safety. The detection of enteric viruses in food is a technical challenge. The complexity and composition of the food matrix, which includes more fats and protein, affect the recovery of the genomic material. In addition, the low level of viral contamination in food and the presence of PCR inhibitory substances in the food matrices affect the molecular detection of viral nucleic acid. Milk includes several components such as fats, casein, whey proteins, and lactose that affect the process of viral nucleic acid extraction and the recovery of enteric viruses [25,26]. The lack of standardized procedures for HEV detection in milk underestimates the risk of HEV infection in milk samples. Consequently, optimizing the methodology of HEV molecular assays in the milk matrix will help to generate more accurate data about the risk of HEV transmission by the contaminated milk.

In this study, we compared the effect of the extraction procedures on the efficiency of the HEV molecular assay. In the whole milk matrix, method A was more efficient than method B in both sensitivity and HEV recovery. One explanation is the use of high-speed centrifuge during the extraction procedure of method A that could help get rid of the fat and other protein in the milk matrix. While method B did not involve the step of high-speed centrifugation. Battistini et al. reported that high-speed centrifugation gave the best method of recovery HAV from the milk matrix [25]. Although the centrifuge speed and time needed could differ, the application of centrifugation in the extraction procedure could affect the recovery of enteric viruses from the milk matrix.

Then we asked if the components of the milk matrix affect the molecular assay for HEV. We did not notice a difference in LoD_95%_ between whole milk matrix, skim milk, and milk serum using method A, but a slight improvement was observed in the HEV recovery rate after the removal of fat and casein proteins suggesting that the protocols used in method A could get rid of the inhibitory substances present in the milk during the procedure steps. On the other hand, a significant improvement in the LoD_95%_ and virus recovery yield was recorded using method B after the removal of both fats and casein protein. In a parallel line, Yavarmanesh et al. reported that the elimination of fat globules and separation of casein micelles from other components in raw milk is the best methodology for the recovery of enteric viruses [26]. While, another study showed that fat was the most facilitating in the recovery of viral RNA from milk, and the casein content of milk was the highest inhibitory factor from the viral recovery from the milk [27]. The discrepancy between the two studies about the effect of fat globules on the viral RNA recovery from the milk is still unclear. However, it could be attributed to the difference in extraction reagents and procedure, the virus recovered, the viral inoculum, the amount of fat to be added, etc. Further studies need to ascertain these points.

Since we achieved a higher sensitive assay (LoD_95%_ 96.34 IU/mL) using method B and milk serum matrix, we asked if this improvement in HEV molecular assay could improve the detection rate of HEV RNA in the milk samples collected from HEV positive animals. Previously we reported that three out of 28 (10.7%) samples were positive for HEV RNA using method A and whole milk matrix [13,14]. Interestingly, reassessment of the same samples using method B in extraction and milk serum matrix, we found that six out of 28 (21.4%) were positive for HEV RNA, probably due to the improved sensitivity (of this assay that could detect a low level of HEV RNA in the milk sample. The viral load in the three positive samples was lower than LoD_95%_ of method A (300 IU/mL) which explained the reason that method A could not detect HEV RNA in these samples.

Then we assessed the effect on the removal of fat on the performance of the ELISA assay. Fats in the milk can affect the absorbance optical density (OD), increase the false-positive results, and reduce the assay specificity [47]. We noticed a significant increase in S/C.O. in the skim milk samples compared to whole milk samples regarding anti-HEV IgG and HEV Ag. The removal of fats from the milk reduces the false positive absorbance in the milk samples, which, in turn, give a more accurate cut off calculation and improve the ELISA performance. This was clearly shown in three whole milk samples in which S/C.O. of anti-HEV IgG were around the cut off value. After the removal of fat from these samples, the result of anti-HEV IgG and HEV Ag was clearer and more precise.

The EFSA described the validated International Organization for Standardization ISO 15216-1:2017 to quantify HAV and human norovirus in several food matrices such as bivalve molluscan shellfish, soft fruit, and stem and bulb vegetables. However, milk-based products were not included in the ISO. A recent study showed that ISO 15216-1:2017 procedure did not result in the best method in HAV detection in the milk-based matrices, probably due to the presence of fats and protein in the milk that could interfere with ISO method [25]. Additionally, Cuevas-Ferrando et al. reported the limited suitability of the ISO 15216-1:2017 concentration procedure for recovering HEV in bottled water [48]. Further studies need to evaluate the effect of ISO method on the concentration of HEV from different milk matrices.

There are some limitations to our study. First, we did not test the effect of HEV concentration from different milk matrices on the efficiency of HEV molecular assay. Second, we did not study the effect of the extraction procedures on milk-based food matrices such as yogurt, ice creams, and cheese. The research on HEV in the milk is recently highlighted, up to our knowledge, there is no report on the detection of HEV in milk-based food products.

This study showed that the HEV molecular assay could be improved by the selection of the extraction procedure that matches with the milk matrix. Method B showed higher performance with milk serum matrix, but not with whole milk or skim milk matrices. In comparison, method A matches with all milk matrices with acceptable LoD_95%_ 300 IU/mL., A previous study showed that the milk matrix affects the performance of the cobas^®^ HEV assay (Roche Diagnostics), which is an automated real-time PCR with LoD_95%_ of 18.6 IU/mL. While a 10% milk matrix did not negatively influence the sensitivity of the Roche cobas^®^ HEV assay, therefore, milk samples diluted 1/10 in PBS were used for molecular detection of HEV [28]. Likewise, Hennechart-Collette reported that the extraction process affects the detection of NoV in dairy products. They compared the effect of homogenization of milk and cottage cheese samples on the recovery efficiency of noroviruses. Homogenization of milk products in TGBE followed by proteinase K enzymatic inactivation on ice increased the recovery efficiencies to 98.87% for NoV GI, and 96.50% for NoV GII compared to homogenization of these products in water followed by proteinase K treatment on ice (76.38%) or homogenization of these products in proteinase K followed by heat treatment at 60 °C (57.14%) [20]. To date, qRT-PCR is the gold standard method for the detection of HEV in human, animal, and environmental samples regarding sensitivity, specificity, reliability, and also availability. The cell culture system for HEV is limited and only certain adapted isolates can grow efficiently in vitro [32]. Additionally, the HEV load present in the milk may be very low, making its replication in vitro more challenging. More recent molecular assay techniques such as a digital PCR and next-generation sequencing could show better performance in the HEV molecular assay.

## 5. Conclusions

This study showed that the HEV molecular assay in the milk samples could be improved by the selection of the extraction procedure and removal of inhibitory substances from the milk, such as fats and casein. Method B showed higher performance with milk serum matrix, but not with whole milk or skim milk matrices. While method A showed acceptable LoD_95%_ and virus recovery yield with all milk matrices. Removal of fats from the milk could improve the HEV serological assays. Enhancement of the molecular and serological assays of HEV will improve HEV detection rate in low level contaminated milk samples and without need for further sample dilution. Additionally, this methodology could be useful for detection of HEV in milk food products.

## Figures and Tables

**Table 1:** Comparison of the effect of matrix and extraction procedure on the molecular assay of HEV.

	Virus Inoculum (IU/mL)	Method A (QIAamp Viral RNA Mini)		Method A (High Pure Viral Nucleic Acid, Roche)		Method B	
		PBS	Milk	PBS	Milk	PBS	Milk
		Pos/Total ^a^	Pos/Total ^a^	Pos/Total ^a^	Pos/Total ^a^	Pos/Total ^a^	Pos/Total ^a^
Positive control stool	3 × 10^5^	3/3	3/3	3/3	3/3	3/3	3/3
	3 × 10^4^	3/3	3/3	3/3	3/3	3/3	3/3
	3 × 10^3^	3/3	3/3	3/3	3/3	3/3	2/3
	3 × 10^2^	3/3	3/3	3/3	3/3	3/3	1/3
	3 × 10^1^	2/3	0/3	2/3	0/3	3/3	0/3
	3	0/3	0/3	0/3	0/3	1/3	0/3
LoD_95%_ of HEV RNA (IU/mL) ^b^		96.6	300	96.6	300	19.4	6.127 × 10^3^
LoD_95%_ of IPC (copies/mL)		64.2	651.8	64.2	651.8	12.9	1412.3

^a^ Pos/total: number of the HEV positive samples/total number of tested samples. ^b^ calculated according to Wilrich, C.; Wilrich, P.-T. J. AOAC Int. 2009.

**Table 2:** Comparison of the cycle threshold (Ct) and virus recovery yield in undiluted RNA and 1/10 diluted RNA extracted by method B.

Virus Stock	Undiluted RNA			1/10 Diluted RNA		
	Pos/Total ^a^	Ct ^b^	Mean HEV Recovery	Pos/Total ^a^	Ct ^b^	Mean HEV Recovery
3 × 10^5^	3/3	26.12 ± 0.35	3.75	3/3	29.34 ± 0.43	2.28%
3 × 10^4^	3/3	29.67 ± 0.21	1.71	1/3	32.31	1%
3 × 10^3^	2/3	32.76 ± 0.51	1.16	0/3	NA	NA

^a^ Pos/total: number of the HEV positive samples/total number of tested samples. ^b^ Ct: cycle threshold, the average Ct of the positive samples ± SD.

**Table 3:** Comparison of the extraction procedures on the sensitivity of HEV RNA detection in different milk matrices.

Method	LoD95% ^a^	Whole Milk Matrix ^b^	Skim Milk Matrix	Milk Serum
Method A (manual extraction)	LoD95% HEV (IU/mL)	300	300	300
	LoD95% IPC (copies/mL)	651.8	200	140.22
Method B (automated extraction)	LoD95% HEV (IU/mL)	6.127 × 10^3^	977 IU/mL	96.34 IU/mL
	LoD95% IPC (copies/mL)	1412.3	140.22	24.98

^a^ Calculated according to Wilrich, C.; Wilrich, P.-T. J. AOAC Int. 2009. ^b^ Data mentioned in Table 1 and presented here again for comparison.

**Table 4:** Evaluation of the effect of extraction procedures and milk components on potential HEV positive milk samples.

Sample	Reference	HEV Markers in Milk (Data from Previous Reports)	Detection of HEV RNA					
			Method A			Method B		
			Whole Milk	Skim Milk	Milk Serum	Whole Milk	Skim Milk	Milk Serum
**Cow milk (n = 8)**	[14]	Sample (n = 1) (+ve for anti-HEV IgG, HEV Ag, and HEV RNA)	+ ^a^ 1/1	+ 1/1	+ 1/1	− ^b^ 0/1	+ 1/1	+ 1/1
		Samples (n = 7) positive for anti-HEV IgG and negative for HEV RNA and HEV Ag	− 0/7	− 0/7	− 0/7	− 0/7	− 0/7	+ (1 out of 7) Titer 2.5 × 10^2^ IU/mL
**Goat milk (n = 20)**	[13]	Samples (n = 2) (+ve for anti-HEV IgG, HEV Ag, and HEV RNA)	+ 2/2	+ 2/2	+ 2/2	- 0/2	+ 1/2	+ 2/2
		Samples (n = 3) (+ve for anti-HEV IgG and HEV Ag and negative for HEV RNA	− 0/3	− 0/3	− 0/3	− 0/3	− 0/3	+ (1 out of 3) Titer 1.87 × 10^2^ IU/mL
		Samples (n = 15) positive for anti-HEV IgG only	− 0/15	− 0/15	− 0/15	− 0/15	− 0/15	+ (1 out of 15) Titer 1.68 × 10^2^ IU/mL

^a^ + means positive for HEV RNA ^b^ – means negative for HEV RNA.
